# Contribution of Clown Therapy in the Training of Psychiatry Residents in Alagoas - Brazil – An Experience Report

**DOI:** 10.1192/j.eurpsy.2026.11974

**Published:** 2026-07-31

**Authors:** L. C. Carneiro, M. I. O. de Morais, A. F. C. Ximenes, L. V. de Araújo, P. F. B. de Araújo, A. B. D. C. Alves, F. O. Barbosa, B. D. A. A. L. Tavares de Melo, V. L. D. Melo-Neto, D. B. B. Ferreira, E. D. F. Correia

**Affiliations:** 1 Universidade Estadual de Ciências da Saúde de Alagoas; 2 Universidade Federal de Alagoas, Maceió, Brazil

## Abstract

**Introduction:**

Laughter is a biological and social reaction that fosters emotional connection, promotes well-being, and highlights the human side of our nature. Clown Therapy leverages laughter as a tool for humanizing care, transforming the often sterile and painful hospital environment into a space of creativity, connection, and therapeutic possibilities.

**Objectives:**

This study aims to explore the redefinition of non-conventional care practices and to examine how playful, humor-based therapies, such as Clown Therapy, can benefit mental health, particularly for psychiatry residents.

**Methods:**

This is an experience report from a Psychiatry resident who incorporated Clown Therapy into her training as part of a medical project in 2019. The report evaluates the impact of this approach on her clinical practice, including patient interactions and personal development.

**Results:**

Clown Therapy significantly influenced the resident’s ability to adapt to psychiatric hospitalizations and the management of psychiatric diagnoses. The use of playful techniques resulted in improved patient rapport, lower levels of anxiety, depressive symptoms, and insomnia during the training period. Additionally, this approach enhanced the resident’s empathy in clinical consultations and fostered a more compassionate approach to psychiatric care.

**Conclusions:**

The findings underscore the value of integrating non-pharmacological strategies, such as Clown Therapy, into psychiatric training and patient rehabilitation. This approach not only aids in the therapeutic relationship but also contributes to the mental well-being of both patients and healthcare providers.

**Disclosure of Interest:**

None Declared

